# ANN-Based Bridge Support Fixity Quantification Using Thermal Response Data from Real-Time Wireless Sensing

**DOI:** 10.3390/s24165350

**Published:** 2024-08-19

**Authors:** Prakash Bhandari, Shinae Jang, Ramesh B. Malla, Song Han

**Affiliations:** 1 Department of Civil and Environmental Engineering, University of Connecticut, Storrs, CT 06269, USA; prakash.bhandari@uconn.edu (P.B.); ramesh.malla@uconn.edu (R.B.M.); 2 Department of Computer Science and Engineering, University of Connecticut, Storrs, CT 06269, USA; song.han@uconn.edu

**Keywords:** frozen bearing, support fixity, artificial neural network (ANN), low-cost wireless sensing network (WSN), bridge joint monitoring, bearing stiffness, finite element modeling, thermal response of bridge

## Abstract

Bridges are critical infrastructures that support our economic activities and daily lives. Aging bridges have been a major issue for decades, prompting researchers to improve resilience and performance through structural health monitoring. While most research focuses on superstructure damage, the majority of bridge failures are associated with support or joint damages, indicating the importance of bridge support. Indeed, bridge support affects the performance of both the substructure and superstructure by maintaining the load path and allowing certain movements to mitigate thermal and other stresses. The support deterioration leads to a change in fixity in the superstructure, compromising the bridge’s integrity and safety. Hence, a reliable method to determine support fixity level is essential to detecting bearing health and enhancing the accuracy of the bridge health monitoring system. However, such research is lacking because of its complexity. In this study, we developed a support fixity quantification method based on thermal responses using an Artificial Neural Network (ANN) model. A finite element (FE) model of a representative highway bridge is used to derive thermal displacement data under different bearing stiffnesses, superstructure damage, and thermal loading. The thermal displacement behavior of the bridge under different support fixity conditions is presented, and the model is trained on the simulated response. The performance of the developed FE model and ANN was validated with field monitoring data collected from two in-service bridges in Connecticut using a real-time Wireless Sensor Network (WSN). Finally, the support stiffnesses of both bridges were predicted using the ANN model for validation.

## 1. Introduction

Bridges are crucial infrastructures of transportation networks that hold a huge economic and social value for nations. However, a significant portion of the global bridge network is reaching the end of its design lifespan. In the USA, more than 42% of bridges are over the age of 50 years [1], and more than 178 million vehicles pass through structurally deficient bridges. Due to budgetary constraints [1], logistical challenges, and a large number of deficient bridges, comprehensive repair and rehabilitation efforts for all deficient bridges may not be feasible in the immediate future. Therefore, monitoring bridge health is becoming vital for prioritizing interventions and ensuring public safety through the timely detection of damage to the bridge. Research in structural health monitoring of bridges has developed advanced technologies and methods for early detection of structural issues in the bridge. However, most of these methods and systems were developed to detect damage to superstructure elements like decks, girders, etc. [2]. These methods often rely on the predefined bridge models with assumed support fixity conditions [3,4], which are most of the time highly idealized and far from the actual condition of bearings. The agencies working on repair and rehabilitation recognized joints and supports as the single biggest reason for bridge deterioration [5,6]. However, there is a lack of proper methods to assess the health of bridge joints and bearings.

Bearings in the bridge are crucial components that transfer the load from the superstructure to the substructure. They are designed to constrain a specific degree of freedom for the superstructure, and based on this, all the superstructure design limits, i.e., strength limits (stresses) and service limits (deflections), etc., are calculated and designed for those limits. For substructure design, only load from the constrained degree of freedom of bearings is considered. Hence, this means the design of all bridge components is made by assuming a predefined restraining behavior of bearings. However, the performance of bearings in in-service conditions was affected by multiple factors like environmental factors, rusting, and lack of lubrication in moving parts, the collection of debris (dirt, sand, and deicing salts) from leaky expansion joints, material degradation due to chemicals like deicing salts, etc. [7,8]. Because those bearings fail to provide movement or increase resistance for movement, restricting the desired movement of the superstructure, these cases are generally referred to as frozen bearings. Although this is considered one of the major issues to be identified and rectified during bridge maintenance by bridge management agencies, it is not widely studied in the literature. The FHWA inspection manual [9] specifically recognizes frozen bearing as one of the major types of damage, along with cracking, corrosion, overloading, and impact damage, that occur in any type of bridge, irrespective of the material or design of the bridge.

A frozen bearing condition in bridges, if not identified and rectified in time, can lead to serious consequences for the overall health of the bridge, potentially even causing it to collapse. Due to freezing, the bearing will not be able to allow superstructure movement for thermal expansion/contraction or traffic loading, leading to overstress of structural elements and potential cracking [10]. In the rocker bearing, when any of the bearings has restricted movement, it is vulnerable to overturning and vertical misalignment of spans in skew bridges [8]. In extreme cases, the horizontal forces generated due to frozen bearings can be sufficient to move or damage bridge piers and substructure components [11]. Because of such serious implications for bridge health, frozen bearing condition assessment is considered a major task during a bridge inspection. Due to the lack of a more accurate empirical or theoretical framework, inspectors have to rely on their subjective judgment based on a visual assessment of the orientation of bearings, the presence of debris and rusts, etc. Consequently, there is a chance of false identification of the problem, as with other visual inspection methods. A data-driven model based on sensor measurements would facilitate more accurate and timely detection of frozen bearing damage and repair of such crucial components.

Currently, there are no methods to directly quantify the stiffness of in-service supports/bearings on bridges. However, a study by Locke et al. [12] presented frozen bearing identification by estimating the stiffness of bearings using a vibration-based modal updating method. They proposed that rotational spring stiffness and the magnitude of friction at the bearing could be used to simulate frozen bearing damage in the bridge. Rotational spring stiffness was determined for this purpose using vibration-based drive-by health monitoring systems. However, for in-service conditions, the modal frequency is affected by superstructure damage or deterioration [13,14] and environmental conditions, such as temperature [15]; it is difficult to pinpoint the exact magnitude of support fixity or frozen bearing condition. Moreover, implementing this method requires determining the bridge’s modal frequency and accurately simulating the entire bridge structure, which will be costly (economically and computationally). Thus, a method based on parameters influenced solely by support fixity (e.g., axial thermal expansion of the bridge) will enable a more precise prediction of bearing freezing and help reduce uncertainty in modal-based damage detection of the superstructure by accurately quantifying support fixity.

In addition, the exact quantification of support fixity level is crucial in vibration-based damage modeling and identification for bridges because the boundary conditions significantly influence the modal properties of the structure. The degree of fixity at the supports affects the stiffness distribution and structural constraints, which impact the natural frequencies, mode shapes, and modal strain energy distribution [16,17]. Variation of support fixity level during the service period is very likely due to factors like soil-structure interaction, bearing degradation, or unintended restraints [17,18]. This change in boundary conditions alters the modal characteristics, potentially masking or amplifying the effects of damage elsewhere in the structure. Inaccurate assumptions of the support fixity level lead to errors in the analytical models used for damage detection algorithms, resulting in misinterpretation of changes in modal parameters, incorrect damage localization, or severity estimation, leading to false-positive or false-negative damage detection [16]. Therefore, a reliable support fixity estimation system and methods are urgently needed.

Wireless sensor networks (WSNs) are a group of spatially distributed sensing and communication devices that are interconnected to monitor physical and environmental conditions like temperature, strain, displacement, and vibration through wireless communication [19]. WSNs offer a cost-effective and scalable solution for bridge health monitoring by eliminating the need for extensive cabling and enabling remote data acquisition from strategically placed sensors [19]. The assessment of bridges using WSNs facilitates the early detection of structural anomalies through continuous monitoring. By taking timely intervention against detected damages, the safety, resilience, and lifespan of the bridge can be enhanced [20].

The WSN can be used for frozen bearing detection due to its aforementioned strengths. The frozen bearing condition or support fixity level can be quantified in terms of the support stiffness value, which can be best determined by measuring the thermal expansion/contraction of the bridge in in-service conditions. Since the quasistatic structural response is primarily governed by temperature variations, the effects of traffic loads can be considered as noise superimposed on the thermal response [21]. Thus, longitudinal displacement measurements using WSN with a low sampling frequency mainly contain a thermal response, based on which support stiffness can be estimated. Compared to modal updating methods based on vibration measurements, static thermal response monitoring provides direct stiffness estimates without complex modal parameter extraction, enabling efficient data collection and transmission over WSNs [22,23]. When there is minimal or no resistance from supports, a bridge will undergo free thermal movement. This movement is directly influenced by the thermal expansion coefficient of the bridge material and the bridge’s length. However, if there are frozen bearings or conditions where the supports offer increased fixity, the thermal movement behavior of the bridge changes significantly. Instead of experiencing simple linear expansion and contraction, the bridge will exhibit restrained thermal movements. The extent of this restrained movement is contingent upon the degree of fixity provided by the supports. Hence, the thermal response measured from bridges will essentially contain support stiffness information and can be quantified with suitable inverse calculation methods, which are known to be challenging due to uncertainties.

The recent development of machine learning algorithms can facilitate complex frozen bearing quantification. Artificial Neural Networks (ANNs) are among the simplest and most versatile machine learning tools available for predictive modeling. With their ability to learn complex nonlinear relationships from data, ANNs can predict the underlying causes or system parameters when provided with the corresponding effects or responses [24]. However, implementing ANNs for structural health monitoring is still challenging due to the need for extensive training data and the potential for overfitting or hasty generalization to unseen scenarios [25]. Additionally, the black-box nature of ANNs can make it difficult to interpret the underlying relationships learned by the network, which may raise concerns regarding transparency and accountability in critical infrastructure applications [26]. Thus, the possibility of generating training by simulating real structures using their digital twins in different scenarios makes ANNs highly promising options for modeling various structural damages.

To this end, many researchers reported that FE structural models have been used to generate training data in many of the studies [21]. A model-free damage detection approach is proposed [24] using ANNs trained on data gathered from simulations of train passages on the finite element model of a fictitious railway bridge. This study used a training data set from an FE simulation of two damage scenarios and a healthy bridge. The ETABS model has been utilized to generate building response data under different seismic loadings to train the ANN model to predict seismic safety parameters [27]. An offshore jacket structure was simulated in ABAQUS to generate training data for the ANN model to extract damage-sensitive features from frequency data [28]. Therefore, an ANN model trained on the results of FE simulations of typical highway bridges could be used to predict bearing stiffness.

This paper develops a bridge support quantification method using an ANN model based on quasi-static bridge displacement responses and temperature data using WSN. For training purposes, a steel girder-concrete deck composite highway bridge is modeled in ANSYS. Various numerical simulations of different support stiffnesses and superstructure damage scenarios were conducted using this model. The longitudinal displacement at the end of the girder was obtained for different thermal loadings (uniform temperature change and thermal gradient as per AASHTO [4]) in the bridges. These data, with Gaussian white noise, are used to train an ANN model. Then, the trained ANN model employed to predict the support stiffness of two in-service highway bridges was predicted: one with visibly healthy elastomeric bearings and another with visibly rusted steel bearings. The field response was monitored using a real-time WSN with an ultrasonic distance sensor and digital temperature and humidity sensors. The result shows the strong potential of this method to relay relevant bridge health monitoring information regarding support to stakeholders for better infrastructure maintenance.

## 2. Methodology

**Figure 1:**
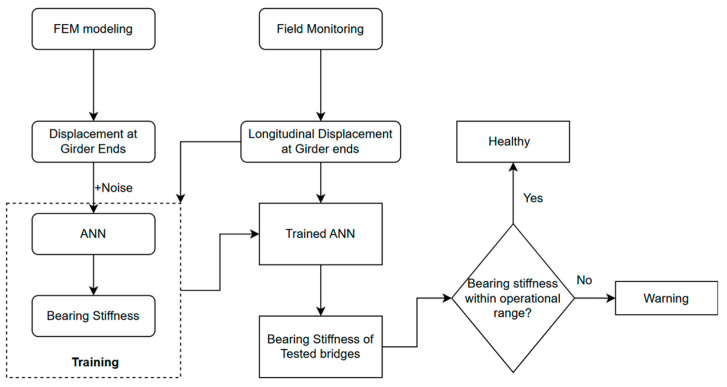
Schematic representation of research methodology.

This study develops a machine learning-based support fixity level (stiffness) quantification method through an FE simulation and field monitoring of in-service highway bridges. A schematic representation of the detailed steps taken to develop the bearing health assessment method is shown in Figure 1. This study consisted of three distinct steps, as follows:

1. FEM modeling and simulation: A FE model of a representative highway girder-bridge with a healthy and damaged superstructure was prepared in ANSYS Mechanical and analyzed under different thermal loading conditions for varying axial stiffness of the bearing using a spring support model.
2. ANN training and modeling: The ANN model has been trained in a MATLAB (R2024a) deep learning package using the numerically simulated thermal response from the FE model. To ensure the model’s robustness in field conditions, the training data was analogous to the field measurement data, and a Gaussian white noise equivalent to that observed in field monitoring was added to the simulation results.
3. Field Monitoring and Validation: Two in-service bridges have been monitored using a low-cost wireless sensor network. The field test measures the longitudinal displacement at the girder ends at a relatively low sampling frequency (0.05 Hz), where the transient effects due to live loads are assumed to appear as noise. The bearing stiffnesses of monitored bridges were predicted using the trained ANN model. The assessment of prediction has been made based on the consistency of prediction and agreeableness of predicted results with visual observation of bearing conditions. When the baseline stiffness level of bearings is known, issues like frozen bearings or bearing failure can be identified based on prediction.

### 2.1. Finite Element (FE) Model

**Figure 2:**
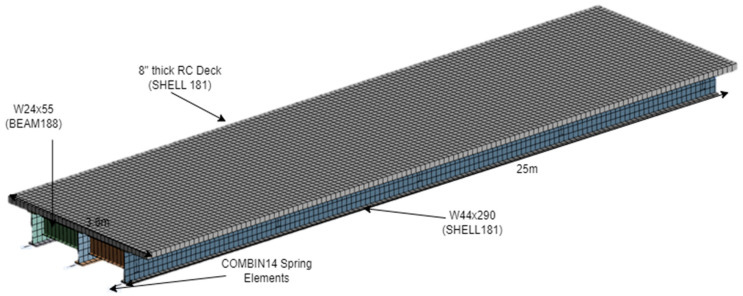
ANSYS model of the bridge with mesh.

A hypothetical yet representative steel girder—reinforced concrete (RC) deck composite highway bridge is modeled using ANSYS (shown in Figure 2). A single span of the bridge with a span of 25 m (82 ft) and a width of 3.6 m (12 ft) supported on 3-AISC W44 × 290 steel sections is considered for this study. The AISC W24 × 55 beams at a spacing of 6 m are used as lateral braces for the longitudinal girders. The geometric dimensions and material type of each structural component used in the simulated bridge are shown in Table 1. These geometric dimensions and sectional properties (geometry and material properties) of the FEM model bridge are those used in the typical design of girder bridges used in Connecticut and are currently in service. Since the effect of railing on the overall thermal response of the bridge is insignificant and adds a significant computational load to the FEM model, the railings are not modeled. The material properties of steel and concrete used for the simulation are shown in Table 2. These values are those recommended in design codes for new construction, and they are assumed to be unaltered during the service period. Bearings are not modeled explicitly; however, their restraint is simulated using the degrees of freedom, longitudinal spring, and varying stiffness.

The longitudinal girders and deck slab are meshed with SHELL181 (4-noded shell element) of size 0.2 m, and cross bracings are meshed with BEAM188 (2-noded Timoshenko beam element). A linear spring is attached to the bottom flange to model a varying axial stiffness of the bearing. The spring is modeled using the COMBIN14 element. The contact between the spring and the flange is created using the CONTA177 and TARGE170 elements. One end of the bridge is considered hinged with no bearing damage (boundary condition: translatory displacements along all x, y, and z-directions, ux = uy = uz = 0; rotation about x and y-axes, ϴx = ϴy = 0). The other end boundary is modeled by the different stiffness of the spring and fully restrained vertical displacement.

The sizes of mesh and element types mentioned above were chosen to provide computationally efficient and reasonably accurate results. A full bridge model using 3D tetrahedral and hexahedral elements was prepared in ANSYS Mechanical and solved in UConn-High Performance Computing (HPC) for a single support stiffness case and used as a reference result. Then, a model using 2D elements was optimized for element types by comparing them. The element size was obtained using a mesh refinement study available in ANSYS Mechanical, and optimal mesh sizes were used for simulation.

### 2.2. Numerical Simulations with FE Model

**Figure 3:**
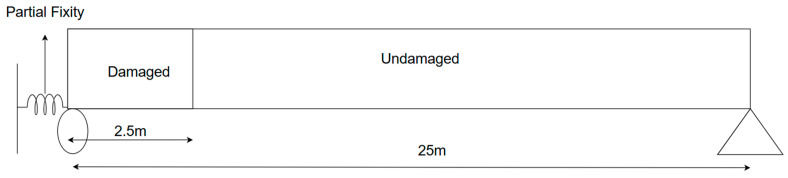
Bearing simulation method and damaged portion of the bridge.

**Figure 4:**
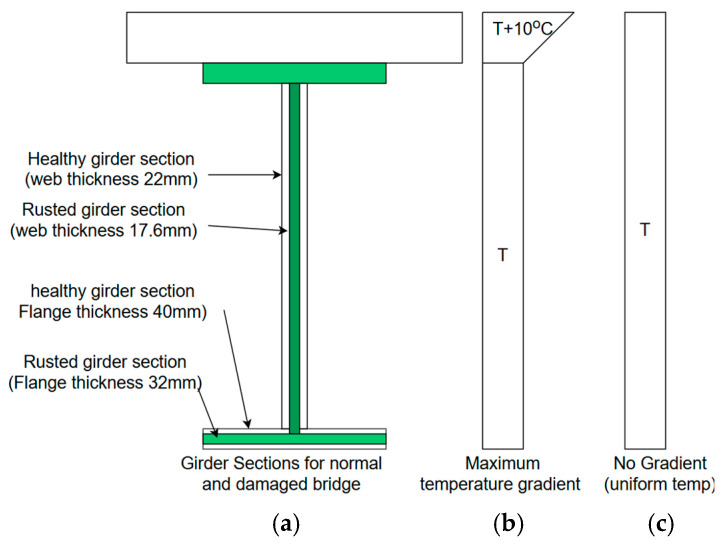
(**a**) Bridge section for damaged (shaded) and undamaged section; (**b**) maximum vertical temperature gradient considered; (**c**) uniform temperature profile.

Using an FE model, the thermal response of the bridge under different temperature variations and distributions is simulated for different structural conditions of the bearing and superstructure. The superstructure damage is simulated as a 10% thickness loss from each face of the bottom flange and web of the main girder up to 2.5-m distance from the end. This damage is based on the field observation of corrosion patterns due to leaks from faulty expansion joints, as shown in Figure 3 (elevation) and Figure 4a (section). The response is calculated for two thermal conditions: (i) uniform temperature change throughout the bridge from −30 °C to 50 °C at the step of 1 °C without considering any temperature gradients (shown in Figure 4c). (ii) A temperature change from −30 °C to 50 °C with a specified maximum temperature gradient as per AASHTO LRFD [29] in the composite bridge, as shown in Figure 4b. For all analysis cases, the reference temperature is assumed to be 10 °C [30], the typical value used in the state of Connecticut.

A healthy superstructure and a damaged superstructure scenario were analyzed for the axial stiffness of bearings varying from 0.5 kN/mm to 10^5^ kN/mm. The bridge, along the vertical direction, was assumed to be supported on rigid supports. The connection between the deck slab and girders was assumed to be rigid. This assumption holds in most cases due to the rigid shear connection between them, unless the shear connection is lost due to deterioration, which causes a change in the rotation of the girder end. Since the ANN model is developed to work with static thermal response data, where the effects of live loads and other transient loads are considered as measurement noise, no other loadings are explicitly simulated. The axial displacement at the top and bottom of the girder under the thermal loading conditions mentioned above was obtained at different axial stiffness levels using the FE model.

**Figure 5:**
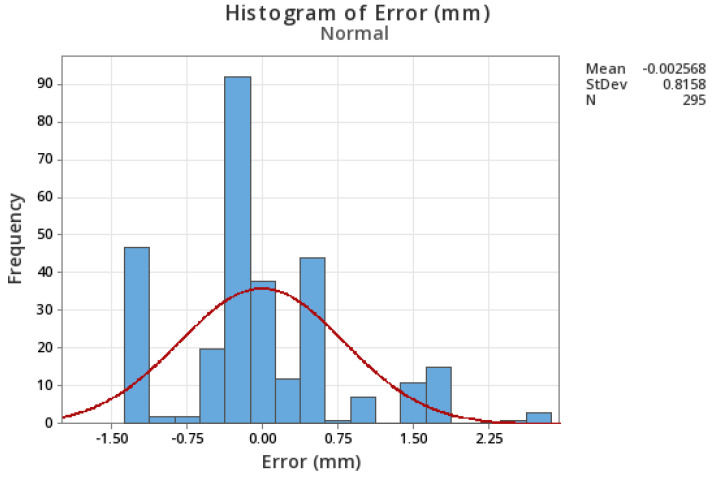
Sensor measurement error histogram.

Field monitoring data inherently contains measurement noise, and its quantification is crucial to incorporate into the ANN model to ensure the model’s robustness and accuracy in predicting the axial stiffness of bridge bearings under in-service conditions. This helps to prevent overfitting to idealized data, enabling the ANN to perform reliably when faced with the inherent uncertainties and variability present in actual sensor measurements. The simulation result is combined with the Gaussian white noise at a level equivalent to that obtained from the field monitoring system. The typical error histogram for the low-cost ultrasonic distance sensor (HC-SR04) is shown in Figure 5. The error is considered normal, with a mean of −0.0025 mm (practically 0) and a standard deviation of 0.81 mm. The root mean square error (RMSE) for this sample was 0.79 mm. Since in the field monitoring, the transient dynamic response of the bridge due to live loads will also appear as noise, its level is expected to be slightly higher. Hence, a Gaussian white noise with a mean of 0 and a standard deviation of 1.25 mm is added to the simulated result to represent the field monitoring data. The noise-laden thermal response data is used as a predictor of bearing stiffness, based on which bearing freezing or bearing failure will be decided.

### 2.3. Artificial Neural Network

The Feedforward ANN (FANN) [31] was employed to establish a model to predict bearing stiffness from quasistatic longitudinal displacement and temperature measurements. ANN is trained on the FE-simulated bridge thermal displacement response using the MATLAB deep learning toolkit [32]. The temperature at the midsection of the girder (T), longitudinal displacement at the top flange of the girder (d_t_), longitudinal displacement at the bottom flange of the girder (d_b_), girder end rotation (θ) as inputs, and bearing stiffness as output. The training datasets, after infusing noise, were further processed to enhance the training stability and convergence of the model. The displacements are made nondimensional by dividing them by the span length of the bridge. The bearing stiffness was converted to a logarithmic scale and then normalized.

**Figure 6:**
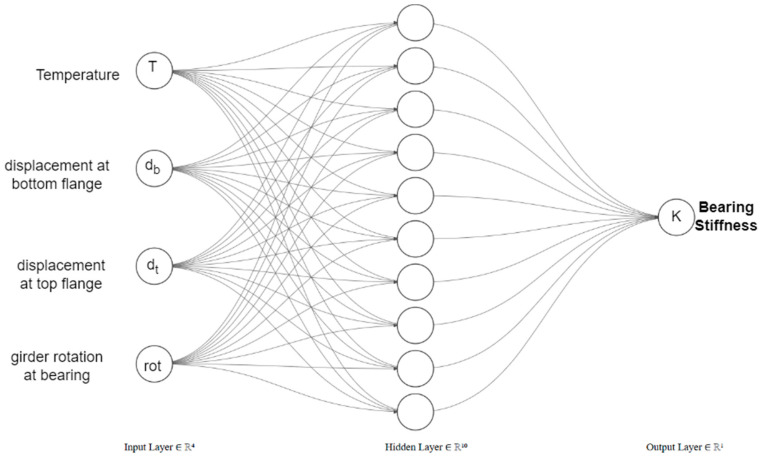
ANN architecture.

The network is trained using the Levenberg–Marquardt (LM) algorithm [33] to combine the benefits of Gauss–Newton and Gradient descent methods. The hyperbolic tangent sigmoid transfer function is used as an activation function for the hidden layer and the linear transfer function for the output layer. The architecture of the neural network was optimized through the trial-and-error process, and the network with a single hidden layer with 10 nodes was found optimal. The hyperparameters for the ANN model included a learning rate of 0.01, a total of 1000 epochs, a batch size of 32, and the use of the Adam (Adaptive Moment Estimation) optimizer. For the evaluation of neural network performance, the mean square error (MSE) metric is adopted. The best performance is obtained for a neural network with one hidden layer having ten nodes, as shown in Figure 6. The total data set of size 8643 was divided into training, validation, and test sets in the proportions of 70%, 15%, and 15%, respectively.

## 3. Field Joint Monitoring Using Wireless Sensor System

The longitudinal displacement and temperatures of two in-service bridges are collected from expansion joints using a wireless sensor network. The ANN model is used to predict the stiffness of the bearings of tested bridges based on data collected from these bridges.

### 3.1. Bridge Testbed

**Figure 7:**
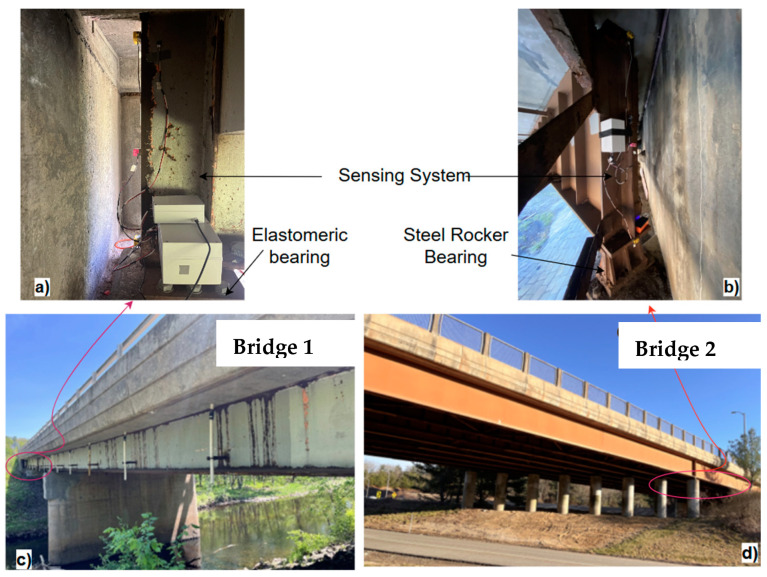
(**a**) Monitoring system deployed in Bridge 1 and the measured gap between girder and back wall; (**b**) monitoring system deployed in Bridge 2 and the measured gap between girder and back wall; (**c**) Bridge 1; (**d**) Bridge 2.

Two steel girder RC deck composite bridges, Bridge IDs: CT01531 and CT02570, with different bearing conditions, were selected from the National Bridge Inventory (NBI) in Connecticut for field monitoring. The testbed bridges are shown in Figure 7. The bridge CT01531 will be referred to as “bridge 1”, and that of CT02570 will be referred to as “bridge 2” in the following section of the manuscript.

Bridge 1, located on the CT-195 route in Coventry, CN, USA, is a 2-span, continuous deck bridge with span lengths of 22.5 m (74 ft) each. It was originally constructed in 1959 and reconstructed in 1993 by adding one more girder to increase the width. The superstructure was inspected in June of 2023, and its superstructure is rated 5 (NBI59); there are no specific comments available for bearings or expansion joints. It has relatively newer elastomeric bearings on one side and steel-pinned rocker bearings on the other. The bridge has asphalt plug expansion joints with a 6″ gap between the abutment’s girders and the back wall. It consists of 6-AISC W36 × 170 girders with 18.5 mm (1.25 inch) thick bottom plates. The expansion joint has not shown any sign of leakage, and there was no visible rusting or damage to the superstructure. The bridge consists of an RC deck of thickness 210 mm (8.25 inch) with a 63.5 mm (2.5 inch) thick asphalt overlay.

Bridge 2 is located over the US-06 route in Windham, CN, USA. This is a 2-span continuous bridge with a total length of 105.75 m (367 ft) and has continuous girders and a deck. It was originally constructed in 1973 and rehabilitated in 1995. The last DoT inspection of this bridge was conducted in August 2022, and its superstructure was rated as 6 (NBI59); however, there are no detailed reports on bearings or expansion joints available. It consists of steel rocker bearings on one end (the monitored side) and pinned rocker bearings on the other side of the abutment. The foam seal expansion joint was highly leaky, and there was significant corrosion of the girder bottom flange and web near the expansion joint (approximately 2.5 m from the end). The bridge was skewed by an angle of 40°, and the gap between the girder end and the abutment back wall is 300 mm (1 ft) at reference temperature. It consists of eight built-in plate girders of depth 1.83 m (6 ft) and a truss bracing system. Since the rusting on the bearing is significant, it was expected to offer higher resistance for longitudinal movement. The summary of the monitoring schedule is shown in Table 3. 

### 3.2. Sensing System

A wireless sensor network (WSN) has been implemented to monitor longitudinal displacement and temperature from the testbed bridges. The longitudinal displacement is measured by measuring the gap width between the girder end and the back wall of the abutment. The HC-SR04 ultrasonic distance sensor [35] measures the distance between the girder end and the back wall at any given time, acting as a sensing component for the network. Additionally, the Digital Humidity Temperature (DHT) 2.0 sensor [36] is employed to measure the temperature of the girder and the humidity levels of the surrounding environment. For robust and efficient wireless communication, the network leverages the 6TiSCH (IPv6 over the Time-Slotted Channel Hopping composed of IEEE 802.15.4e) protocol, ensuring reliable data transmissions with low power consumption and high resilience to interference [37]. Data collected from the field sensors are routed through a gateway hosted on a Raspberry Pi 4, which acts as an intermediary, collecting sensor data from the microcontroller and forwarding it to the internet. The central server for data storage and real-time data visualization is hosted on Amazon Web Services (AWS) EC2 [38], providing scalable, and secure cloud infrastructure. The details of these components, including all configurations, hardware features, schematics, sensor specifications, installation, and calibration of the sensing system, can be found in an article by Fils et al. [34].

## 4. Results and Discussion

This section presents and discusses thermal response results from FE simulations, ANN model training results, thermal response from field monitoring, and predicted bearing stiffness results of monitored bridges.

### 4.1. Thermal Response of the Bridge

**Figure 8:**
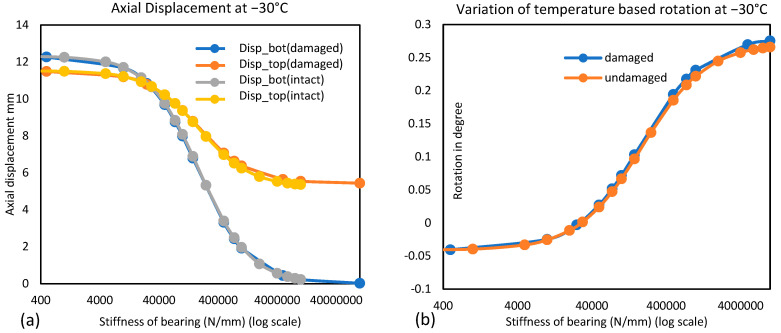
(**a**) Longitudinal thermal displacements for different support stiffness (at −30 °C); (**b**) typical rotation behavior of damaged and healthy superstructures at −30 °C.

**Figure 9:**
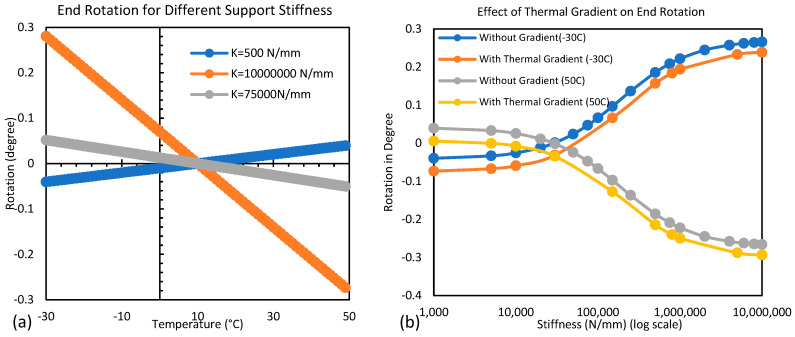
(**a**) Girder end rotation at different temperatures for different support stiffness; (**b**) girder end rotation for different support stiffness at two different temperatures of girder −30 °C and 50 °C with and without vertical thermal gradient.

The displacement response of the composite bridge under different temperature distributions in conditions with different damage scenarios of the superstructure is studied based on the results of finite element simulation. Figure 8a shows the longitudinal displacement of the bridge at the top and bottom flange levels for damaged and undamaged superstructures with different stiffnesses of support. This shows that the damage to the superstructure has a negligible effect on the longitudinal thermal movement of the bridge. The presence of damage marginally increases displacement at the top of the structure. However, the displacement at the bottom flange level gets marginally reduced due to damage. This constitutes a small increase in the rotation or flexural curvature of the bridge due to damage, as shown in Figure 8b. This difference in rotation between damaged and undamaged superstructures increases slightly as support becomes stiffer. This can be attributed to the fact that longitudinal movement is dependent on the length of the superstructure and the thermal expansion coefficient. The difference in thermal movement along the depth of the superstructure is due to the fixity of the bearings. Figure 9a supports this; as support stiffness increases, rotation increases. When the temperature of the bridge changes uniformly, the movement near the bottom fiber of the beam is restricted by support, but the effect of this restriction gets reduced along the height of the superstructure, causing almost free thermal movement near the top. This causes a flexural curvature, and hence, rotation at the end of the girder is observed. The curvature of the bridge depends upon the sectional properties and elastic modulus of the superstructure; any change in these will affect the rotation. In this case, since damage is considered in terms of section loss due to corrosion near support, rotation was affected slightly due to damage.

In Figure 9a for K = 500 N/mm, rotation below neutral temperature (10 °C) is negative or counterclockwise, but as stiffness increases to 75,000 N/mm, this rotation becomes positive/clockwise. This demonstrates that uniform temperature change causes rotation in the opposite directions if support does not provide fixity in longitudinal movement (low support stiffness). Thus, for a bridge, the girder rotation will be zero for a given value of longitudinal stiffness of the bearing, e.g., in this case, the girder rotation will be zero for some stiffness value between 500 and 75,000 N/mm. This may be due to the difference in thermal expansion coefficients of steel and concrete (approximately 20%), causing ‘thermal bowing’ due to differential thermal expansions. 

The effect of the thermal gradient on the composite bridge depends on the support stiffness and the overall temperature. Figure 9b shows the typical rotational behavior under a vertical thermal gradient at two representative temperatures (one freezing and another expanding).

For low support stiffness, during the low-temperature time (see −30 °C curve in Figure 9b), steel contracts more as compared to concrete due to ‘thermal bowing’, and the presence of a thermal gradient (high temperature on top) exacerbates this bowing by further reducing concrete contraction. Hence, it increases the negative bending/rotation (hogging). However, during the high-temperature time (see 50 °C curves in Figure 9b), steel expands more than concrete without a thermal gradient, but the presence of a thermal gradient helps the concrete to expand more, reducing the ‘thermal bowing.’ Hence, positive bending/rotation is reduced, as shown in Figure 9b. For high support stiffness, during the low-temperature time (see −30 °C curves), the presence of a thermal gradient reduces the positive bending/rotation (sagging). This is due to the lower contraction of the top of the superstructure, which has a higher temperature. However, during high-temperature times (see 50 °C curves in Figure 9b), the presence of a thermal gradient increases the negative bending/rotation (hogging). This is due to the higher expansion of the top of the superstructure, which has higher temperatures. Thus, the presence of the specified thermal gradient reduces the positive bending but enhances the negative bending.

### 4.2. ANN Model

**Figure 10:**
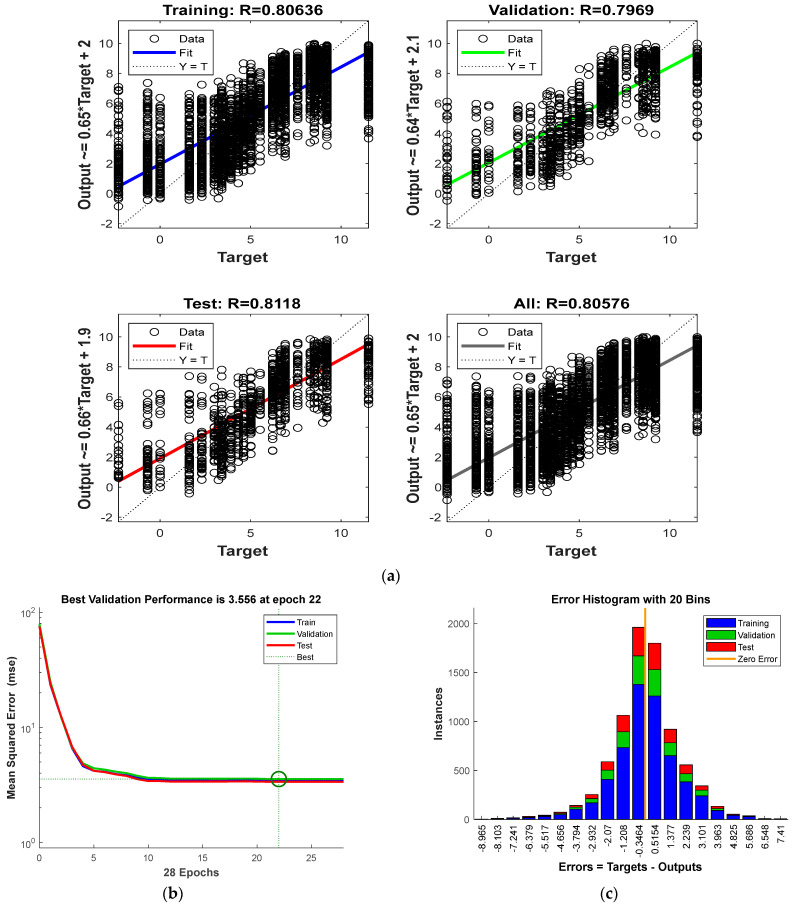
(**a**) Predicted vs. actual support stiffness (in log scale) scatter plots in different data sets; (**b**) training progress/convergence history in terms of MSE; (**c**) prediction error histogram (stiffness in the log of kN/mm scale).

ANN was trained using the dataset from undamaged and damaged cases separately. The training history and model prediction accuracy of either dataset do not differ in significance. Thus, a final ANN model is trained using a combined data set of thermal responses from superstructure-damaged and undamaged bridges. Similarly, another two datasets, one response with a thermal gradient and a response without a thermal gradient, were also trained. The *R*-value is a measure of the ANN’s prediction accuracy and is the correlation coefficient between the predicted value and the target value of the bearing stiffness. The *R*-value for the ANN model without a temperature gradient was 0.80, and that for the model with a temperature gradient was 0.78. Since the overall model weightage and accuracy are not different, these two data sets were also combined. Hence, a final ANN model with all possible thermal response cases is trained, and training accuracy for each test, validation, and training set is presented in Figure 10a. The training progress is shown in Figure 10b, and the error histogram is presented in Figure 10c.

The model achieved its optimal validation performance with a mean squared error of 3.446 (in base 10 logarithmic of kN/mm stiffness scale) or 2792.5 kN/mm of real stiffness at epoch 22. This indicates that the training data is sufficiently adequate for the model to learn and generalize well to unseen data before overfitting begins. The ANN model demonstrates strong predictive capability for support stiffness, with *R*-values of 0.80636 for the training set, 0.7969 for the validation set, and 0.811 for the test set, resulting in an overall *R*-value of 0.8057. The prediction equations, such as 0.65 × Target + 2 (ideally 1 × Target), indicate consistent and reasonably accurate estimations of the target variable. Additionally, the error histogram shows an almost normal distribution, with a mean of approximately 0 and a standard deviation of 0.52 (less than 3.5 kN/mm). These results collectively suggest that the model is robust enough to handle any thermal gradients and superstructure damages, generalizes well, and is a reliable tool for predicting support stiffness.

### 4.3. Field Joint Monitoring

The gap opening between the girder end and the abutment back wall is measured using an ultrasonic distance sensor through the wireless sensing system explained in the previous section. The thermal expansion/contraction is obtained by subtracting the gap opening from the reference temperature (10 °C). Due to the high noise in the sensor near the top flange, its readings are not considered in this study. The thermal displacement response of both monitored bridges is presented below.

#### 4.3.1. Bridge 1

**Figure 11:**
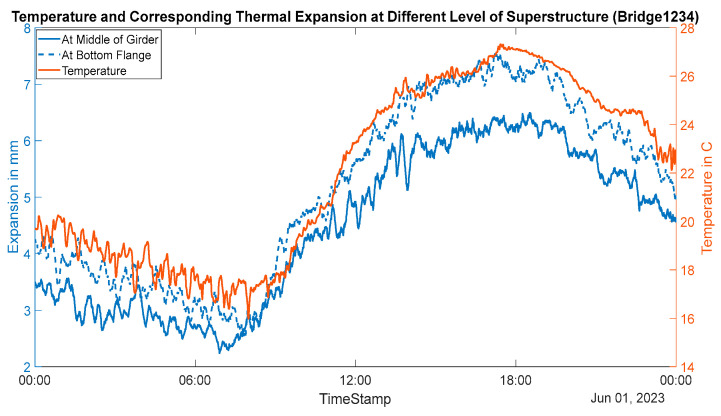
Thermal movements at different girder sections (Bridge 1).

**Figure 12:**
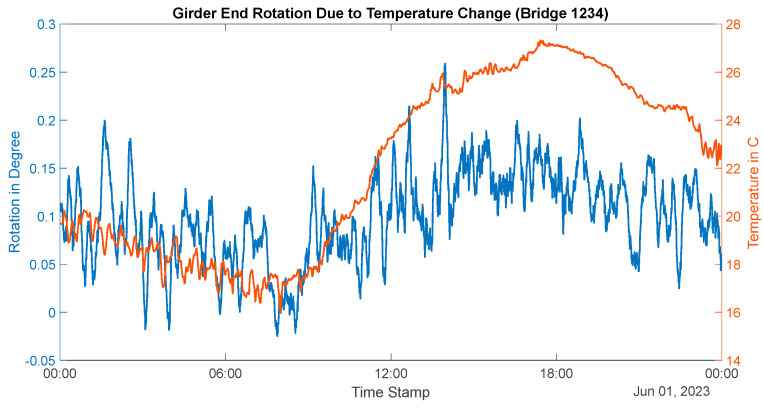
Thermal rotations at the girder end (Bridge 1).

This bridge was tested during the summer, and the girder’s temperature varied from 14 °C in the early morning to a maximum of 27 °C at approximately 5 PM. The variation of temperature, along with corresponding thermal expansion at different sections of the girder, is shown in Figure 11. The range of thermal movement was found to be 2.5 mm to 5.5 mm at approximately the middle of the girder and 2.5–7.4 mm (7.6 mm free linear thermal movement) at the bottom flange of the girder. This expansion behavior indicates a relatively small value of support stiffness and ‘thermal bowing’ of the bridge. This is most likely to be accurate because this bridge has an elastomeric bearing at the monitored end. The rotation due to this thermal bowing is presented in Figure 12. This shows a rotation of approximately 0.02°–0.17° during the daily temperature fluctuation cycle.

#### 4.3.2. Bridge 2

**Figure 13:**
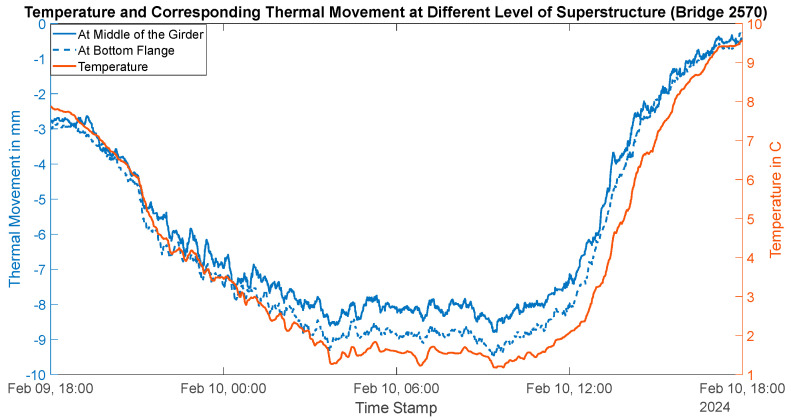
Thermal movement at different girder sections (Bridge 2).

**Figure 14:**
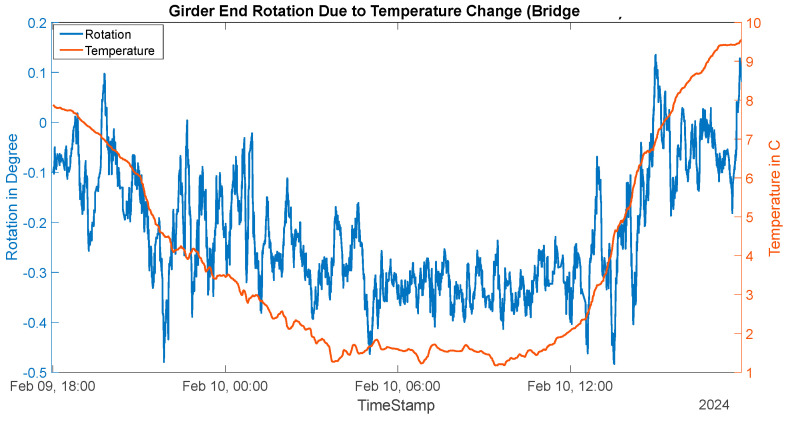
Thermal rotation at girder end (Bridge 2).

Thermal movement of this bridge is conducted during the winter because of the high likelihood of bearing freezing due to accumulated dirt and high corrosion on its steel bearings. The girder temperature was found to vary from 1 °C to 10 °C. The day’s temperature variation and corresponding thermal contractions are shown in Figure 13. The bridge contracted up to 9.5 mm at the bottom flange level and up to 8.5 mm at the middle of the girder (free linear contraction of steel 11.34 mm). This shows that the bearing resistance is higher in this bridge as compared to Bridge 1. Here, the bridge is contacting 83% of free steel contraction as compared to 97% of free steel contraction in Bridge 1. Additionally, the girder end rotation varied from −0° to −0.4° (shown in Figure 14), indicating higher thermal bending.

### 4.4. ANN Predicted Support Stiffness

The support stiffness corresponding to the field monitoring results for both of these bridges is predicted using the ANN model. For Bridge 1, the predicted support stiffnesses were almost uniform with a very low value (mean 9.73 kN/mm), and those for Bridge 2 were found to change with temperature with a relatively higher value (from 250 kN/mm to 1000 kN/mm).

**Figure 15:**
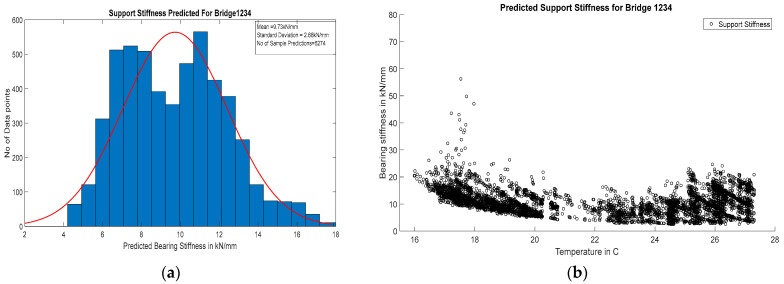
(**a**) Histogram with normal distribution fit of support stiffness for Bridge1234; (**b**) scatter plot temperature vs. corresponding predicted bearing stiffness for Bridge1234.

For Bridge 1, the stiffness was uniform throughout the day, irrespective of changes in temperature. Figure 15a shows a normal distribution of predicted stiffness with a very low standard deviation of 2.68 kN/mm. The scatter plot shown in Figure 15b shows the temperature independence of bearing stiffness throughout the monitoring period and temperature range. The predicted results align with the visual inspection inferences from the bridge. The bearing was elastomeric, which has very low lateral stiffness, and since the bearing was relatively newer with no signs of deterioration, it was free from dust. The variance in predicted stiffness is due to the presence of noise in the measurement. The outliers near 17 °C are caused by anomalous events like ultrasonic noise from the environment or erroneous sensor readings. However, since the temperature during the monitoring period was not near freezing, there may be chances of freezing when the temperature reaches near zero.

**Figure 16:**
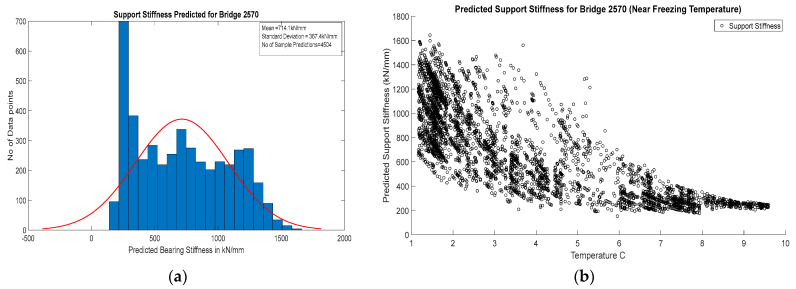
(**a**) Histogram with normal distribution fit of support stiffness for Bridge 2570; (**b**) scatter plot temperature vs. corresponding predicted bearing stiffness for Bridge 2570.

**Figure 17:**
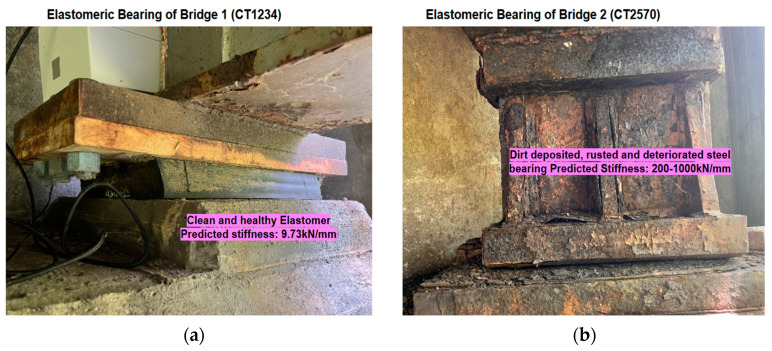
(**a**) Healthy looking elastomeric bearing in Bridge 1; (**b**) deteriorated bearing of Bridge 2.

For Bridge 2, the predicted stiffness was found to vary from 200 kN/mm to 1600 kN/mm, with a mean value of 714.1 kN/mm. Figure 16a shows a histogram plot of predicted stiffness across the measured temperature. The predicted support stiffness has very weak normality with a very high standard deviation of 367.4 kN/mm. This lack of normality in the prediction is explained by the dependence of support stiffness on temperature, as shown in Figure 16b. This shows an exponential increase in bearing stiffness as temperature goes below 6 °C; above this temperature, bearing stiffness is constant at approximately 300 kN/mm. The high variability prediction is higher at lower temperatures, which may be due to the time-dependent physical or chemical changes in bearings or material deposited in the moving planes near the freezing temperatures. Above 6 °C, prediction results show higher prediction precision. These results indicate that there may be a ‘Frozen bearing’ condition in the bridge. In the absence of any technique for quantifying bearing stiffness, the calculated bearing stiffness values were assessed based on the visual appearance and qualitative assessment of bearing conditions. The visual appearance of this bearing is shown in Figure 17b. There is the deposition of dirt in the moving part of this bearing, along with heavy rusting and leaking from the expansion joint, justifying the relatively higher stiffness predicted by the ANN model.

These two case study bridges show that the ANN model trained in this study is able to predict support stiffness with reasonable accuracy, even with thermal response data measured from noisy, low-cost sensors. Thus, by integrating this ANN model with a low-cost WSN system, bearing stiffness can be predicted in real-time. The real-time alert system can be developed by defining lower and upper limits of stiffness values. Additionally, this method can be implemented along with a vibration-based damage detection system to quantify support fixity levels for higher precision in superstructure damage detection. Since this method is based on an ANN model trained on FE-simulated data, proper measurement, and quantification of the noise level in the field monitoring data need to be performed. This might turn out to be a challenging task because of the effects of traffic loadings and environmental conditions. Moreover, the computational intensiveness of the ANN model might make it difficult to integrate with a real-time monitoring system, which typically consists of low-capacity hardware components. However, advancements in sensing systems and the computational power of hardware components will help make this method more useful and robust.

## 5. Conclusions

In this paper, the thermal response of a typical steel girder concrete deck composite highway bridge is studied using numerical simulation on the FE model; an ANN model based on those thermal responses is developed for the prediction of bearing stiffness. From the numerical simulation of thermal loadings, it has been found that uniform temperature changes cause the superstructure to bend, with the nature and magnitude of this bending influenced by support stiffness, beam flexural stiffness, and temperature. It was observed that section loss due to corrosion in the superstructure has a negligible effect on the longitudinal expansion and contraction of the bridge. Additionally, the presence of a thermal gradient mitigates positive bending (sagging) due to uniform temperature changes while exacerbating negative bending (hogging). Field monitoring of in-service bridges confirmed superstructure rotation due to thermal bowing and support restrictions. The ANN models trained on data sets containing thermal gradients or superstructure damage have shown that the support stiffness prediction is not affected by such cases. The support stiffness predicted by the ANN model was found to be consistent with the visual judgment of the bearing condition, indicating the usefulness of the model. Longitudinal thermal movement is the simplest bridge response, influenced only by the thermal expansion coefficient of the bridge material, bridge geometry, and bearing stiffness. Since the thermal expansion coefficient and geometrical parameters are typically unaffected by bridge operational conditions or deterioration and are precisely known, by monitoring longitudinal thermal movements and using an Artificial Neural Network (ANN) model, bearing stiffness can be accurately predicted for any type of bridge. This ANN model allows engineers/researchers/bridge management agencies to diagnose bearing health conditions and associated problems, including “Frozen bear-ing”, based on its stiffness estimation by measuring thermal movement data using low-cost sensing systems. This enables timely maintenance actions that enhance the safety and durability of the bridge. This model will also be a valuable tool to eliminate uncertainty related to support restraints from the conventional vibration-based damage detection methods, facilitating more accurate damage localization and quantification of the superstructure.

## Figures and Tables

**Table 1:** Geometry and materials used in the FE model.

Element	Geometry	Material
Deck	8″ thick	Concrete
Main Girders	W44 × 290	Steel
Cross bracings	W24 × 55	Steel

**Table 2:** Material Properties and Constants used in simulation.

Parameter	Steel	Concrete
Modulus of Elasticity	200 Gpa	25 Gpa
Poisson’s Ratio	0.3	0.2
Coefficient of Thermal Expansion	1.2 × 10^−5^/°C	1 × 10^−5^/°C
Density	7850 kg/m^3^	2400 kg/m^3^

**Table 3:** Monitoring Schedule.

Description	Bridge 1	Bridge 2
Start of Monitoring	1 June 2023 00:00:00	9 February 2024 18:00:00
End of Monitoring	1 June 2023 23:59:59	10 February 2024 18:00:00
Season	Summer	Winter
Monitoring System	6TiSCH-based WSN [34]	6TiSCH-based WSN [34]
Data Retrieval	Real-time Remote	Real-time Remote
Sampling Frequency	0.06 Hz	0.05 Hz

## Data Availability

The authors will make the raw data supporting this article’s conclusions available upon request.
